# MiracleNet: A Biologically Interpretable Machine Learning Model for Resected Non-small-cell Lung Cancer

**DOI:** 10.34133/csbj.0145

**Published:** 2026-07-14

**Authors:** Rashika Jakhmola, David A. Selby, Mert Cihan, Dusan Prascevic, Elisabetta Petracci, Paola Ulivi, Enriqueta Felip, Rocío Caro-Consuegra, Franco Stella, Piergiorgio Solli, Desideria Argnani, Milena Urbini, Johannes U. Mayer, Sebastian J. Vollmer, Christian Martin, Jan Ewald, Maximilian Sprang

**Affiliations:** ^1^ Department of Dermatology, University Medical Center of the Johannes Gutenberg University, 55131 Mainz, Germany.; ^2^ Data Science & Its Applications, German Research Center for Artificial Intelligence (DFKI), Kaiserslautern, Germany.; ^3^ Technical University of Munich, Munich, Germany.; ^4^ Goethe University Frankfurt, 60629 Frankfurt am Main, Germany.; ^5^ Department of Computer Science, University of Kaiserslautern–Landau (RPTU), Kaiserslautern, Germany.; ^6^ Faculty of Biology, Johannes Gutenberg University, Mainz, Germany.; ^7^ Center for Scalable Data Analytics and Artificial Intelligence (ScaDS.AI) Dresden/Leipzig, Leipzig University, Leipzig, Germany.; ^8^ Unit of Biostatistics and Clinical Trials, IRCCS Istituto Romagnolo per lo Studio dei Tumori (IRST) “Dino Amadori”, Meldola, Italy.; ^9^ Biosciences Laboratory, IRCCS Istituto Romagnolo per lo Studio dei Tumori (IRST) “Dino Amadori”, 47014 Meldola, FC, Italy.; ^10^ Thoracic Tumors Group, Vall d’Hebron Institut d’Oncologia (VHIO), Vall d’Hebron Barcelona Hospital Campus, Barcelona, Spain.; ^11^ Department of Oncology, Vall d’Hebron Hospital Universitari, Vall d’Hebron Barcelona Hospital Campus, Barcelona, Spain.; ^12^ Thoracic Surgery Department, AUSL Romagna, Forlì, Italy.; ^13^ Thoracic Surgery, Fondazione IRCCS Istituto Nazionale dei Tumori, Milan, Italy.; ^14^ Thoracic Surgery Department, AUSL Romagna, Ravenna, Italy.; ^15^ Research Center for Immunotherapy (FZI), University Medical Center of the Johannes Gutenberg-University Mainz, 55131 Mainz, Germany.; ^16^ Institute of Quantitative and Computational Biosciences, Johannes Gutenberg University, Mainz, Germany.

## Abstract

Lung cancer remains one of the leading causes of cancer-related mortality worldwide. Accurate prediction of relapse is notoriously difficult, posing substantial challenges to patient care and necessitating advanced tools to improve prognostic outcomes. MicroRNA (miRNA) expression profiles hold promise as biomarkers for predicting relapse, yet existing predictive models lack interpretability or sufficient predictive performance. Biologically informed neural networks have emerged as a modeling approach incorporating biological interpretability and predictive accuracy. Here, we introduce MiracleNet, to our knowledge the first visible neural network in which sparse connectivity is structured by the miRNA → target gene → pathway hierarchy for disease-free survival prediction from circulating miRNA in non-small-cell lung cancer and the first to expose interpretable importances jointly at all 3 biological layers, with nodes connected by prior knowledge about miRNA targets and related biological pathways. Our model, which also integrates clinical data, achieves a maximum concordance index of 0.76, demonstrates improved generalization over unconstrained neural networks of the same dimensionality (including both dense and sparse architectures lacking biological knowledge), and provides explicit biological interpretability. Our model also highlights several important biomarkers in the form of predictive miRNAs and connected biological pathways. We additionally evaluate MiracleNet under a nested repeated 80/20 protocol, augmented with patient sex and tumor stage as clinical covariates and combined across circulating free and extracellular-vesicle-associated miRNAs through early and intermediate fusion; these analyses are reported as separate sections.

## Introduction

Non-small-cell lung cancer (NSCLC) is a leading cause of cancer-related mortality worldwide, with 85% of all lung cancer cases being attributed to NSCLC [1,2]. Early-stage non-small-cell lung cancer (ES-NSCLC) accounts for 20% to 30% of all NSCLC cases and is marked by a high postsurgical survival rate. Nevertheless, some patients undergo disease relapse, and this probability is highly dependent on tumor stage. However, clinical outcomes can vary among patients within the same disease stage, indicating that other factors may influence the risk of recurrence. Currently, there are no precise and validated methods to classify patients based on their relapse risk [3,4].

MicroRNAs (miRNAs) are a class of small, noncoding RNA molecules that play an important role in the posttranscriptional regulation of gene expression. They are involved in the fine-tuning of cellular processes by binding primarily to the 3′ untranslated regions of target messenger RNAs (mRNAs), leading to mRNA degradation or translational repression, thereby serving as a regulatory mechanism between transcription and translation, providing insights into regulatory networks that may not be apparent from other single-omics data (i.e., mRNA or proteomics alone). Their regulatory function makes miRNA essential in various biological processes, including development, differentiation, proliferation, and apoptosis [5,6].

miRNAs have been proposed as possible diagnostic and prognostic biomarkers [7,8] in NSCLC [9]. For example, down-regulation of the miRNAs *miR-144*, *miR-451*, and *let7f* has been shown to be associated with worse prognosis [10–12]. Moreover, in contrast to other biomarkers relying on tumor tissue samples, miRNAs are detectable in the bloodstream, stemming from either free circulation or extracellular vesicles, making them highly accessible and stable circulating biomarkers [13].

Within the European multicenter MIRACLE project (A Machine Learning Approach to Identify Patients with Resected Non-Small-Cell Lung Cancer with High Risk of Relapse), we aim to develop a biologically informed deep learning model that leverages miRNA-derived data to predict disease-free survival (DFS) in ES-NSCLC and to identify patients at a high risk of relapse. Additionally to circulating free miRNA (cf-miRNA), we explore the miRNAs found in extracellular vesicles (extracellular-vesicle-associated miRNAs [ev-miRNAs]) in the blood of the patients [12,14], taken before resection of the tumor.

Because our model is biologically informed, it offers greater interpretability than a classical neural network, and its structured design enables robust performance even on smaller patient cohorts. In this study, we analyzed a cohort of 189 resected NSCLC patients with available circulating miRNA measurements, including both cf- and ev-miRNAs.

## Related Literature

Incorporation of prior knowledge into deep neural networks is a general concept applied in many domains, like physics or biology [15,16], and has been proposed to increase not only explainability but also performance through inductive bias and regularization.

A recent review of biologically informed neural networks [17] identified applications of pathway-constrained deep learning architectures to miRNA data: Lemsara *et al.* [18] proposed a model called PathME, a pathway-based autoencoder integrating gene expression, miRNA, DNA methylation, and copy number variation data; Lan *et al.* [19] introduced DeepKEGG, a cancer recurrence prediction model combining mRNA, miRNA, and single-nucleotide polymorphism data from The Cancer Genome Atlas with a pathway-based self-attention mechanism. PathME relied on miRBase [20] to obtain predicted target genes for each miRNA, which were in turn mapped to National Cancer Institute (NCI) pathways; by contrast, DeepKEGG used the mirPath server [21] to obtain miRNA sets that are directly associated with Kyoto Encyclopedia of Genes and Genomes pathways. In this work, we used an approach similar to PathME of mapping miRNAs to pathways indirectly via target genes; the mapping was obtained considering multiple regulation databases as outlined in the methods section, similar to Cihan *et al.* [6]. In contrast to PathME, which embeds these mappings inside an autoencoder for multi-omics integration, MiracleNet encodes the miRNA → gene → target → pathway hierarchy as a sparse-mask visible neural network trained directly for DFS and exposes interpretable importances at each of the 3 biological layers on the same forward pass.

Non-omics data, such as patient demographic characteristics (e.g., age and sex) or routinely collected clinical data, are not mappable to genetic pathways. Integrating such data in a biologically informed neural network model requires fusion with pathway embeddings at some point (early, intermediate, or late fusion). Nguyen *et al.* [22] examined the effect of integrating such non-omics (drug) embeddings with genotype embeddings at different stages, finding that late fusion can result in worse performance in a drug response prediction task. However, earlier results on multi-omics data fusion found that early fusion within different omics modalities tends to perform worse than intermediate or late fusion [23], leaving an inconclusive indication whether to perform early or late fusion.

## Methods

### Data

The MIRACLE project is a multicenter European study funded by ERA PerMed JTC2021 (ERP-2021-23680708 – ERP-2021-ERAPERMED2021-MIRACLE). The retrospective cohort was enrolled between 2018 and 2021 within the ERA-NET TRANSCAN-2 (JTC 2016)-RESTING project (ERP-2016-23671110) [14].

The study cohort comprised 189 patients diagnosed with stage I to IIIA NSCLC who underwent surgical resection at the Istituto Romagnolo per lo Studio dei Tumori “Dino Amadori”-IRCCS (Meldola), S. Orsola-Malpighi Hospital (Bologna), and Vall d’Hebron Hospital/Vall d’Hebron Institut d’Oncologia (Barcelona). A peripheral blood sample was collected prior to tumor resection. DFS was defined as time from surgery to recurrence or death, whichever occurred first. The last follow-up update was performed on 2024 January 15.

DFS information was available for 183 of 189 patients, with 59 observed DFS events. Median DFS was not reached (NR; 95% confidence interval [CI]: 53.1 to NR). The median follow-up time was 46.2 months (95% CI: 44.1 to 47.9), estimated using the reverse Kaplan–Meier method.

Plasma samples were processed to extract cf-miRNAs and ev-miRNAs as previously described [14], following standardized operating procedures. After preprocessing and quality control, normalized cf-miRNA expression profiles were available for 169 patients, while ev-miRNA expression profiles were available for 163 patients. All patients included in the cf- and ev-miRNA modeling cohorts had available DFS time, event status, and age information. The cf-miRNA modeling cohort comprised 169 patients with 43 DFS events, and the ev-miRNA modeling cohort comprised 163 patients with 42 DFS events.

Demographic and clinical variables, including age at surgery, sex, and pathological stage, were collected from electronic case report forms using the OpenClinica system. Pathological stage was grouped into 3 categories (stage I, stage II, and stage IIIA) for descriptive analyses.

Prior to model training, miRNA expression features were standardized using *z*-score normalization (zero mean and unit variance). Patient age was normalized separately using the same procedure. The same preprocessing pipeline was applied consistently across all model evaluations.

The multimodal cf + ev fusion analyses introduced in the “Multimodal cf + ev integration” section use the subset of patients with paired cf- and ev-miRNA measurements (*n* = 156, 41 DFS events). The clinical covariate analyses introduced in the “Clinical covariate models” section (age + sex + stage) use the subset of patients with nonmissing sex and stage annotations: *n* = 155 (41 events) for cf-miRNA and *n* = 152 (40 events) for ev-miRNA. The smaller cohorts are due to patient-level missingness; we report cohort sizes alongside each new result so that comparisons remain transparent. The baseline patient characteristics for the present cohort are summarized in Table 1; Kaplan–Meier DFS analyses on the same patient population, including stratification by clinical and pathological stage, are reported in detail in our prior publication on the RESTING cohort (Non-invasive prognostic markers for Resected Early-STage NSCLC: role of circulatING and exosomal miRNAs and free circulating DNA) [14] and are therefore not duplicated here.

**Table 1.** Baseline characteristics of the MIRACLE patient cohort with complete clinical annotation (*n* = 181). The cf-miRNA (*n* = 169) and ev-miRNA (*n* = 163) analytic cohorts used throughout the article are subsets of this clinically annotated cohort. Median follow-up is the reverse Kaplan–Meier estimate reported in the “Model performance” section (data subsection); other quantities are summarized from the RESTING clinical record.

Variable	*n* (%) or median (IQR)
Total patients	181
Age, years (median, IQR)	70 (63–75)
Age, range	44–84
Sex, male	108 (59.7%)
Sex, female	73 (40.3%)
Pathological stage IA	89 (49.2%)
Pathological stage IB	31 (17.1%)
Pathological stage IIA	9 (5.0%)
Pathological stage IIB	19 (10.5%)
Pathological stage IIIA	33 (18.2%)
Histology, adenocarcinoma	147 (81.2%)
Histology, squamous cell carcinoma	34 (18.8%)
Smoking, never ^a^	25 (13.8%)
Smoking, former ^a^	104 (57.5%)
Smoking, current ^a^	41 (22.7%)
Smoking, missing	11
ECOG performance status 0 ^a^	132 (72.9%)
ECOG performance status 1 ^a^	38 (21.0%)
ECOG performance status 2 ^a^	7 (3.9%)
ECOG, missing	4
Adjuvant chemotherapy, yes	28 (15.5%)
Adjuvant chemotherapy, no	147 (81.2%)
Adjuvant chemotherapy, missing	6
Adjuvant radiotherapy, yes	9 (5.0%)
Adjuvant radiotherapy, no	166 (91.7%)
Adjuvant radiotherapy, missing	6
Median follow-up, months	46.2
DFS events	58 (32.0%)

MIRACLE, A Machine Learning Approach to Identify Patients with Resected Non-Small-Cell Lung Cancer with High Risk of Relapse; miRNA, microRNA; cf-miRNA, circulating free miRNA; ev-miRNA, extracellular-vesicle-associated miRNA; RESTING, Non-invasive prognostic markers for Resected Early-STage NSCLC: role of circulatING and exosomal miRNAs and free circulating DNA; IQR, interquartile range; ECOG, Eastern Cooperative Oncology Group; DFS, disease-free survival

^a^ Coding for smoking (0, 1, and 2) interpreted as never/former/current and for ECOG (Eastern 0, 1, and 2) as the standard performance status scale.

### Model architecture

**Fig. 1.**
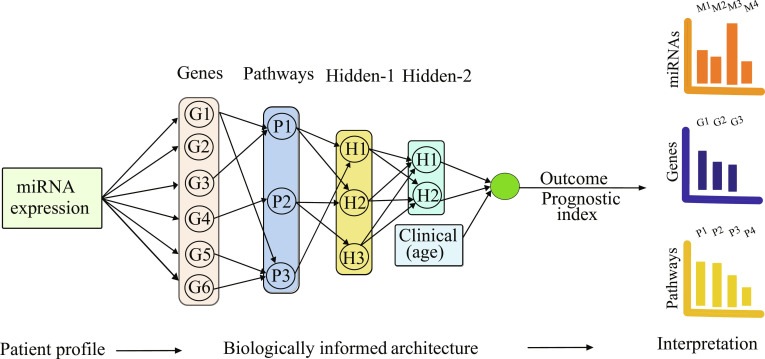
Architecture of the MiracleNet deep learning framework. MicroRNA (miRNA) expression values are mapped to hidden nodes representing gene targets with learnable weights. These nodes, in turn, are mapped to nodes representing biological pathways in Reactome. This is (optionally) followed by 2 densely connected hidden layers, which are finally integrated with clinical data observations to yield a prognostic index for survival prediction. Normalized node importances from the input (miRNA) and 2 biologically informed layers (genes and pathways) may be used to identify possible biomarkers.

Building upon the principles of MiNet [24], we developed MiracleNet, a biologically informed hierarchical neural network [15] designed to predict survival outcomes in NSCLC patients. The model architecture follows a structured miRNA–gene–pathway hierarchy to enhance both interpretability and predictive performance. Two MiracleNet variants were considered: one in which pathway-level representations are directly connected to the output layer and a second variant in which pathway representations are followed by additional densely connected hidden layers. In both variants, clinical information is intentionally introduced only at the final stage of the network. Specifically, in the pathway-only model, clinical covariates are concatenated with pathway-level activations immediately before the Cox proportional hazards output layer, whereas in the deeper variant, clinical covariates are concatenated with the final hidden layer before the output. This design ensures that the network first learns biologically meaningful relationships among miRNAs, genes, and pathways, without early reliance on clinical variables. Figure 1 shows the described model architecture.

### Integration of miRNA–target and pathway information

To integrate biological information into the model architecture, miRNAs were mapped to their target genes using experimentally validated interactions from several publicly available databases. These included DIANA-TarBase v8 [25], miRTarBase [26], TRANSFAC [27], and TargetScanHuman 8.0 [28]. Only interactions that met strict thresholds were included to ensure meaningful selection of functionally relevant and evolutionarily conserved interactions. Specifically, this included positive experimental validation for direct and indirect interactions, complemented by predicted miRNA–target interactions from TargetScanHuman 8.0 [28]. Additionally, pathway information was obtained from the Molecular Signatures Database, which included the Reactome database [29].

### Training and evaluation

Models were trained within a Cox proportional hazards framework to predict DFS. The final output layer estimates a prognostic index as a linear combination of learned biological representations and selected clinical covariates. Importantly, the absence of a bias term ensures adherence to the Cox proportional hazards formulation, and model parameters were optimized by minimizing the negative partial log-likelihood.

Tumor stage is a well-established prognostic factor in NSCLC; however, its inclusion substantially reduced the number of usable samples due to missing annotations. Moreover, as the primary aim of this study is to investigate liquid-biopsy-based prognostic modeling in a minimally invasive or prediagnostic setting, tumor stage would not be available at prediction time. Including stage would therefore introduce a strong postdiagnostic feature that could dominate other inputs and obscure the contribution of circulating miRNA signals. For these reasons, tumor stage was intentionally excluded from the model. Patient age was included as a continuous clinical covariate, as it is routinely available at the time of liquid biopsy collection and does not restrict cohort size. Age was modeled as a real-valued input and was not discretized or dichotomized.

Model training and evaluation were performed using 2 complementary strategies. Hyperparameter optimization was conducted using 5-fold cross-validation on the training data, with each fold using 80% of the data for training and 20% for validation. Hyperparameters, including the learning rate and $L_{2}$ regularization strength, were optimized using grid search based on the mean validation concordance index across folds. Models were trained for up to 500 epochs, with early stopping based on validation loss improvements, and $L_{2}$ regularization was applied to mitigate overfitting. To enforce biological interpretability, sparse connectivity patterns were maintained throughout training.

Final model performance was assessed using 10 independent stratified 80/20 train–test splits, and the reported results reflect the mean and standard deviation of test-set performance across these splits. Stratification preserved the distribution of survival events and time to event by using a composite stratification label derived from event status and discretized survival time. Performance was evaluated using the concordance index (C-index). Computational efficiency was also a key consideration, as the biologically constrained model demonstrated a heavily reduced training time compared to fully connected alternatives.

All models were implemented in Python version 3.12.12 using the PyTorch deep learning framework 2.9.0. The full software dependencies are documented in the accompanying code repository.

#### Nested evaluation protocol

To obtain a conservative estimate of generalization performance, we additionally evaluated selected MiracleNet variants using a nested repeated train–test protocol. Each model variant was evaluated over 10 stratified outer 80/20 splits. Within each outer training partition, hyperparameters (learning rate and $L_{2}$ regularization strength) were selected exclusively via inner 5-fold cross-validation; the outer test partition was held out from all model selection and used only for final scoring. Stratification preserved the joint distribution of event status and discretized survival time. Reported values are the mean and standard deviation of the outer test C-index over the 10 outer splits, together with a 95% CI interval for the mean (normal approximation) and the best observed outer test C-index. The original repeated 80/20 protocol used in the main model performance section above is retained for the unimodal baselines and for the biomarker discovery analyses that depend on those models; the nested protocol is used wherever new analyses are reported.

### Clinical covariate models

To assess the contribution of routinely collected clinical information, we trained additional MiracleNet variants in which patient age, sex (encoded as a binary variable), and tumor stage were appended at the final fusion layer, immediately before the Cox output. Sex was added because it is routinely collected in the electronic case report forms and could in principle contribute prognostic information. Tumor stage was included as a clinically established prognostic benchmark, allowing us to evaluate whether miRNA-derived representations provide complementary information when combined with routinely available clinical predictors. Two stage encodings were evaluated. The first used clinical stage in 3 categories (I, II, and IIIA), and the second used pathological stage in 5 categories (IA, IB, IIA, IIB, and IIIA), both encoded as one-hot indicators. The biologically informed miRNA–gene–pathway path of the network was unchanged in these variants; only the clinical input vector differed. The age-only setting remains the primary configuration because it best reflects a minimally invasive liquid biopsy scenario in which detailed pathological staging may not yet be available.

### Multimodal cf + ev integration

To test whether cf- and ev-miRNA carry complementary prognostic information, we evaluated 2 multimodal fusion strategies on the subset of patients with paired cf and ev measurements (*n* = 156, 41 events). Early fusion concatenates cf- and ev-miRNA features into a single input vector before the biologically informed miRNA–gene layer; the gene and pathway representations are then shared across both modalities. Intermediate fusion processes cf and ev through 2 parallel biologically informed branches, each applying the same gene-to-pathway connectivity mask to its own compartment, and concatenates the pathway activations from the 2 branches before the Cox output layer. Both architectures use age as the sole clinical covariate, so that the comparison isolates the effect of combining the 2 miRNA compartments from the effect of adding clinical information.

### Comparison to differential expression analysis

A differential expression analysis was performed to benchmark the MiracleNet feature rankings against a classical statistical approach. Using limma [30], a linear model was fitted to the normalized miRNA expression matrix with DFS event status and patient age as covariates. Moderated *t*-statistics were obtained via empirical Bayes shrinkage (eBayes), and *P* values were adjusted for multiple testing using the Benjamini–Hochberg procedure. miRNAs with an adjusted *P* value below 0.05 were considered differentially expressed.

In a complementary step, univariate Cox proportional hazards models were fitted for each miRNA individually, with DFS as the outcome and patient age as an additional covariate, using the survival package in R [31]. miRNAs with a nominal P<0.05 for the miRNA coefficient were retained as survival associated. The final set of classically identified biomarkers was defined as the intersection of the limma-significant and the Cox-significant miRNA sets, thereby selecting features that are both differentially expressed between event groups and individually prognostic for DFS.

All analyses were conducted in R (version 4.4.2) using the limma and survival packages.

### Functional enrichment analysis of identified features

To annotate functional associations of miRNAs with known biological processes, we employed precursor miRNA overenrichment analysis for the set of the top 25 features identified either from cf- or ev-miRNAs using the miEAA webserver. [32] We further included annotated TAM 2.0 functions through the rba_mieaa_enrichment function in the R package rbioapi.

## Results

### Model performance

We compared 2 biologically informed MiracleNet variants to 3 non-biological baselines: a sparse neural network, a fully connected neural network of the same depth and width, and a penalized linear Cox proportional hazards model. Model performance predicting patient survival was assessed using the C-index. For all models, final performance was evaluated over 10 independent stratified 80/20 train–test splits, and we report the mean and best test C-indices.

**Fig. 2.**
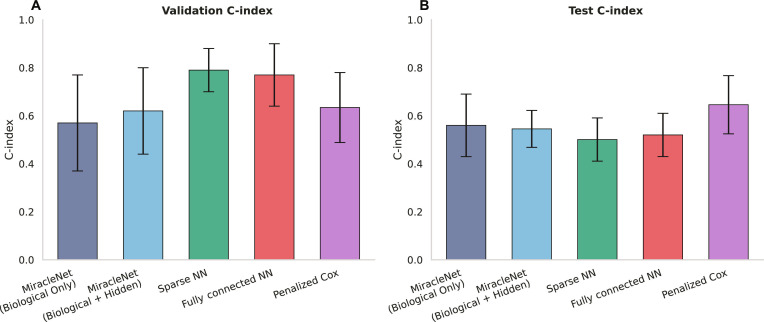
Validation and test concordance index (C-index) of different models on the ev data. (A) Mean validation C-index across 5-fold cross-validation; (B) mean test C-index across 10 random 80/20 train–test splits. Error bars indicate ±1 standard deviation. Biologically informed MiracleNet variants (Biological Only and Biological + Hidden) show slightly lower validation performance than unconstrained sparse and fully connected neural networks but achieve better test-set generalization and provide explicit biological interpretability.

On the validation folds (5-fold cross-validation), the unconstrained sparse and fully connected networks achieved the highest C-indices (Fig. 2A), while the biologically informed MiracleNet variants attained slightly lower validation performance. This pattern is expected: flexible architectures with many free parameters can more easily overfit the training folds of a small clinical cohort. However, strong validation performance in this setting does not necessarily translate to improved generalization on unseen data.

Consequently, when evaluated on test data across 10 random splits, this ranking reverses (Fig. 2B). The biologically informed MiracleNet models seem to generalize better than the unconstrained neural networks, which might be an indication that the biological information enables the network to learn more easily from sparse data than an unconstrained sparse network.

In the ev-miRNA cohort, the pathway-only MiracleNet (Biological Only) and the extended MiracleNet with 2 additional dense layers (Biological + Hidden) achieved mean test C-indices of 0.56 ± 0.13 and 0.54 ± 0.07, respectively (Table 2). In contrast, the sparse and fully connected networks reached lower mean test C-indices of 0.50 ± 0.09 and 0.52 ± 0.09, respectively, despite their stronger validation performance. This discrepancy between validation and test performance indicates substantial overfitting in the unconstrained architectures, whereas the MiracleNet variants exhibit more robust generalization, comparable to the linear baseline.

**Table 2.** Test-set performance on the ev-miRNA cohort across 10 random 80/20 train–test splits. Values denote mean test C-index ± standard deviation and the best test C-index observed over the 10 runs.

Model	Mean test C-index (10 runs)	Best test C-index
MiracleNet (Biological Only)	0.56 ± 0.13	0.76
MiracleNet (Biological + Hidden)	0.54 ± 0.07	0.65
Sparse neural network	0.50 ± 0.09	0.59
Fully connected neural network	0.52 ± 0.09	0.69
Penalized Cox linear baseline	0.65 ± 0.12	0.80

miRNA, microRNA; ev-miRNA, extracellular-vesicle-associated miRNA; C-index, concordance index

The penalized Cox model achieved the highest mean test C-index across runs (0.65 ± 0.12), underscoring the competitiveness of a well-regularized linear baseline on limited data. Yet this model operates directly on the input features and does not expose intermediate miRNA–gene–pathway representations. In contrast, MiracleNet comes close to the average performance of the Cox model, but unlike the linear baseline, it also provides interpretable weights at each biological layer. It allows us to examine how miRNAs propagate through genes and pathways. The best-performing run of the pathway-only MiracleNet reached a test C-index of 0.76, demonstrating that the biologically constrained architecture can achieve strong predictive performance in individual splits.

Performance on the cf-miRNA cohort shows similar tendencies across models and is reported in Table S1.

### Nested evaluation of unimodal MiracleNet models

To ensure separation of hyperparameter selection from final test scoring, we additionally re-evaluated the pathway-only MiracleNet models on both compartments using the nested repeated 80/20 protocol described in the “Training and evaluation” section. On the ev-miRNA cohort, the pathway-only MiracleNet achieved a mean nested test C-index of 0.546 ± 0.070 (95% CI [0.503, 0.589], best run 0.648). On the cf-miRNA cohort, it achieved 0.0485 ± 0.086 (95% CI [0.432, 0.539], best run 0.647). The ev nested mean is very close to the value reported in Table 2 (0.56), but the nested protocol produces a tighter standard deviation (0.070 *vs.* 0.13), consistent with the expectation that strict separation of hyperparameter selection from final scoring yields more stable performance estimates. Accordingly, interpretation focuses on the mean outer test performance and its variability across splits, while the best observed split is reported only as contextual information.

These nested numbers are consistent with the original protocol on the ev cohort and do not change the qualitative ranking of MiracleNet relative to the unconstrained neural baselines reported above. Together, these results indicate that the original performance estimates were not driven by overlap between hyperparameter selection and final test scoring.

We also note an asymmetry between the 2 compartments: in the ev-miRNA compartment, the biologically informed mask delivers a clear generalization advantage over the unconstrained sparse baseline (cf. Table 2), while in the cf-miRNA compartment, it does not (cf. Table S1). We interpret this asymmetry as biologically meaningful rather than as a deficiency: ev-derived miRNAs are reported to be more stable and more enriched for tumor-derived signal than cf-miRNAs [33], and our results are consistent with the hypothesis that a pathway-based biological prior is most informative when the input is already enriched for the biology that the prior describes. The deeper MiracleNet variant (Biological + Hidden in Table 2) trails the pathway-only model on mean test performance; we attribute this to the additional dense parameters diluting the biological prior on a cohort of this size rather than to dense hidden layers being inherently harmful, and we expect the deeper variant to recover on larger cohorts.

### Clinical covariate models

To benchmark the model against a clinically realistic setting, we trained MiracleNet variants that augment the age-only input with patient sex and tumor stage. Two stage encodings were evaluated as described in the “Clinical covariate models” section in Methods. Results are summarized in Table 3 for ev-miRNA and Table S2 for cf-miRNA.

**Table 3.** Nested test-set performance of MiracleNet variants with additional clinical covariates on the ev-miRNA cohort. The age-only model uses the full ev cohort (*n* = 163, 42 events); the variants with sex and stage use the subset of patients with nonmissing sex/stage annotations (*n* = 152, 40 events).

Clinical input	Mean test C-index	95% CI	Best run
Age only	0.546 ± 0.070	[0.503, 0.589]	0.648
Age + sex + clinical stage	0.600 ± 0.082	[0.549, 0.650]	0.751
Age + sex + pathological stage	0.575 ± 0.117	[0.502, 0.647]	0.744

miRNA, microRNA; ev-miRNA, extracellular-vesicle-associated miRNA; C-index, concordance index; C-index, concordance index; CI, confidence interval

In the ev-miRNA cohort, augmenting age with sex and clinical stage raised the mean nested test C-index from 0.546 ± 0.070 to 0.600 ± 0.082 (95% CI [0.549, 0.650]), with the best of the 10 outer splits reaching 0.751; this comparison spans cohorts of slightly different sizes (*n* = 163 age-only, *n* = 152 with clinical covariates) because patients with missing stage or sex annotations were excluded from the latter. The pathological-stage encoding, with 5 rather than 3 categories, performed similarly on the best run (0.744) but with higher variance across splits (±0.117); we attribute this to the smaller per-category sample size of the more granular encoding. In contrast, on the cf-miRNA cohort (*n* = 169 age-only; *n* = 155 with clinical covariates), the addition of sex and stage did not improve mean performance: the clinical-stage variant moved from 0.485 ± 0.086 to 0.459 ± 0.107, and the pathological-stage variant reached 0.553 ± 0.097 (best 0.71). This asymmetry mirrors the pattern seen for the age-only models: clinical covariates appear to compose with the ev-miRNA signal in a constructive way, while in the cf-miRNA compartment, they do not stabilize the model further.

The ev + sex + clinical stage configuration is the best MiracleNet variant in our experiments. Its mean (0.600) approaches the penalized Cox baseline reported in Table 2 (0.65 ± 0.12). A like-for-like comparison would require evaluating a Cox baseline augmented with the same clinical covariates under the nested protocol. A direct comparison with a penalized Cox model using the same clinical covariates under the nested protocol would further clarify the relative contribution of model class versus clinical information and should be considered in future validation work.

### Multimodal cf + ev integration

On the paired subset of patients with both cf and ev measurements (*n* = 156, 41 events), early fusion of cf- and ev-miRNAs into a shared biologically informed network achieved a mean nested test C-index of 0.562 ± 0.066 (95% CI [0.521, 0.602], best run 0.678). Intermediate fusion at the pathway level achieved 0.544 ± 0.061 (95% CI [0.506, 0.581], best run 0.672). Both multimodal variants used age as the sole clinical covariate, making them directly comparable with the age-only unimodal nested models reported in the “Nested evaluation of unimodal MiracleNet models” section, with the caveat that the multimodal cohort is slightly smaller than either unimodal cohort due to patients with measurements in only one compartment.

Early fusion improves modestly over both age-only unimodal models: +0.077 over the cf-only model (mean 0.485) and +0.016 over the ev-only model (mean 0.546), although the CIs overlap and the difference relative to ev-only should be interpreted cautiously. Intermediate fusion at the pathway level did not show a similar improvement, suggesting that the benefit of combining the 2 compartments is captured earlier in the biological hierarchy than at the pathway level. Neither multimodal configuration reached the performance of the ev + sex + clinical stage model (0.600). These findings suggest that in the present cohort, cf + ev integration provides limited additional prognostic benefit beyond ev-miRNA alone, whereas clinical covariates contribute more substantially to performance.

### Biomarker discovery

In addition to improving predictive performance, the biologically structured MiracleNet model enables meaningful biomarker interpretation by design. In the version of the model without hidden layers, the direct connection between miRNAs and survival outcomes allows us to examine input weights to identify important molecular features and connections to target genes and pathways.

For the cf dataset, among the top 25 miRNAs ranked by normalized magnitude of their learned weights, 6 miRNAs were also identified as statistically significant in classical differential expression analysis followed (*mir-101*, *mir-107*, *mir-16*, *mir-29a*, *mir-424*, and *mir-9*), suggesting a concordance between deep learning model based and traditional approaches. Similarly, for the ev dataset, 4 miRNAs emerged in both the top 25 miRNAs by MiracleNet weights and in differential expression analysis (*mir-181a*, *mir-182*, *mir-183*, and *mir-532*).

#### miRNAs

A substantial number of miRNAs appeared in the top 25 lists of both cf and ev models, indicating a shared relapse-associated regulatory signature detectable across liquid biopsy compartments. The intersection between cf and ev included 14 miRNAs: *miR-9*, *miR-203a*, *miR-16*, *miR-107*, *miR-424*, *miR-101*, *miR-17*, *miR-142*, *miR-140*, *miR-195*, *miR-15a*, *miR-340*, *miR-30e*, and *miR-93*. In addition to these overlapping features, each dataset contributed distinct signals: the cf model emphasized *miR-497*, *miR-132*, *miR-330*, *miR-145*, *miR-29a*, *miR-128*, *miR-7*, *miR-30d*, *miR-1271*, *miR-143*, and *miR-29b*, whereas the ev model prominently ranked *miR-22*, *miR-532*, *miR-182*, *miR-96*, *miR-186*, *miR-183*, *miR-15b*, *miR-181a*, *miR-32*, *miR-181d*, *miR-30a*, and *miR-30b*. These dataset-specific patterns can reflect either biological or technical differences between cf- and ev-miRNAs (see Table S3).

#### Target genes of miRNAs

We leveraged MiracleNet to identify not only the most important input features (miRNAs) but also the most important target genes comprising the second layer of the biologically informed neural network. The top 25 target gene features exhibited substantial overlap between cf- and ev-based models. Highly ranked genes included core oncogenic regulators such as *NRAS*, *KRAS*, *MAPK1*, *MAPK3*, *FGF2*, *PIK3CA*, *GRB2*, and *SOS1*, as well as the ubiquitin-related genes *UBA52*, *UBB*, and *UBC*. Additional frequently selected genes included *PPP2CA*, *PTPN1*, and *AKT3* and proteasome-associated components such as *PSMB5*, *PSMD1*, and *PSME3* (see Table S4).

These findings align with canonical NSCLC biology, in which activation of the RAS–MAPK and PI3K/AKT signaling cascades, as well as dysregulation of the ubiquitin-proteasome system and proteostasis regulators, is a widely established contributor to tumor biology [34,35]. The strong reproducibility of gene-level rankings across cf and ev inputs underscores that MiracleNet identifies stable and biologically grounded molecular signatures (see Biological Relevance of Important Features for a discussion on the biological relevance of important features).

#### Target pathways of miRNAs

Analogously to target genes, we identified as well the most important target pathways of input features (miRNAs). The top-ranked pathways showed similarly strong consistency between cf and ev models, too. Notable shared pathways included multiple FGFR-related modules (signaling by FGFR in disease, downstream signaling of activated FGFR1/2/4, and FRS-mediated FGFR signaling), EGFR-associated pathways (EGFR signaling in cancer and signaling by ligand-responsive EGFR variants in cancer), as well as pathways related to DNA replication and checkpoint regulation (CDK-mediated phosphorylation and removal of CDC6, G2/M DNA damage checkpoint, and mitotic metaphase and anaphase) (see Table S5).

Further relevant pathways included stress- and immune-related signaling such as NF-κB activation, the TLR4 cascade, and interleukin-2 family signaling, as well as microenvironmental and angiogenesis modules including VEGFR2-mediated vascular permeability and VEGF signaling. Metabolic and transcriptional pathways such as glycolysis, SUMOylation pathways, and the transport of mature mRNAs derived from intronless transcripts were also prominent.

Across all 3 biological layers—miRNAs, genes, and pathways—MiracleNet identifies coherent, reproducible, and biologically meaningful features associated with relapse risk. The convergence of top-ranked features across cf and ev models supports the robustness of the architecture and illustrates the utility of pathway-constrained neural networks for transparent biomarker discovery.

## Biological Relevance of Important Features

**Fig. 3.**
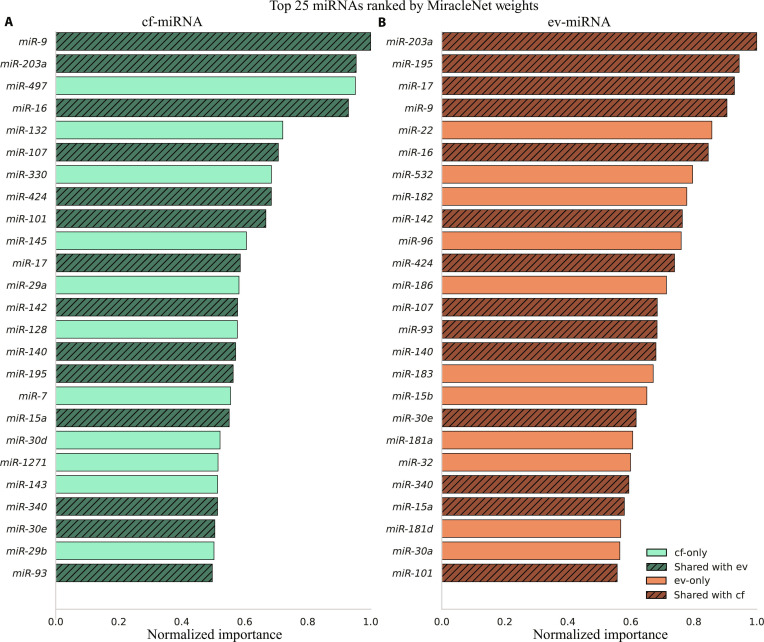
Top 25 most important microRNAs (miRNAs) ranked by normalized importance scores from the MiracleNet model, shown separately for the cell-free (A) and extracellular vesicle (B) datasets. Shared features are highlighted.

The top 25 cf- and ev-miRNAs ranked by MiracleNet weights are presented in Fig. 3. To allow a fair comparison between cf and ev importance values, the MiracleNet importance scores were min–max normalized within each dataset before visualization. Since the overlap with differential expression analysis was low, we further validated these miRNAs by cross-referencing them with established databases using miEAA (including pathway, tissue, and disease reference sets) [32].

For the ev-miRNAs, 23 out of the 25 top-ranked features were significantly overrepresented in NSCLC (adj. P<0.01). Further, these miRNAs showed significant enrichment across 9 functional categories, including apoptosis, immune response, cell cycle regulation, and tumor cell chemosensitivity. For the cf-miRNAs, 21 out of the 25 top-ranked features were similarly significantly overrepresented in NSCLC (adj. P<0.01). Functional enrichment analysis revealed significant associations with 3 biological processes, including apoptosis and immune response. Detailed enrichment results and functional term annotations for the top 25 cf- and ev-miRNAs are provided in Table S6.

Mechanistically, miRNAs contribute to NSCLC through 2 main routes: they can be up-regulated, thereby suppressing tumor inhibitors, or down-regulated, which releases oncogenic drivers. MiracleNet captures both modes. For instance, *miR-17* has been described as promoting cell proliferation by targeting the tumor suppressor SIK, thereby disrupting metabolic checkpoint control and supporting tumor growth. Notably, *miR-17* has also been detected as a circulating biomarker in NSCLC patients [36,37]. Similarly, *miR-9* facilitates epithelial–mesenchymal transition and metastasis by down-regulating TGFBR2 and CDH1, resulting in activation of protumorigenic signaling pathways [38,39]. In addition, *miR-93* exerts oncogenic activity by directly regulating the tumor suppressors *PTEN* and *RB1*, thereby promoting the activation of proliferative and survival pathways in NSCLC [40].

Conversely, several MiracleNet miRNAs map onto tumor-suppressive circuits in NSCLC. *miR-145* has been linked to inhibition of the cell proliferation through targeting of c-Myc and MUC1 [41,42]. Further, its reduced expression has been associated with poor patient survival. Moreover, *miR-101* has been described as inhibiting tumor progression through epigenetic regulation and interference with the AKT signaling pathway, leading to reduced proliferation [43]. A similar suppressive effect has been reported for the *miR-29* family, which constrains Wnt/β-catenin signaling through epigenetic regulation of the Wnt inhibitor WIF-1, resulting in the suppression of tumor growth and induction of apoptosis [44].

Altogether, these findings show that miRNAs identified by MiracleNet not only are statistically enriched for NSCLC but have also been described as mechanistically involved in key cancer hallmarks. This points at biologically informed network models as potential discovery methods, as they integrate regulatory layers across miRNAs and their target genes and pathways to capture the complexity of NSCLC progression and relapse.

## Discussion

In this study, we introduce MiracleNet, a biologically informed neural network designed to predict DFS in resected ES-NSCLC using miRNA expression profiles derived from cf- and ev-miRNAs. By embedding prior biological knowledge through a structured miRNA–gene–pathway architecture, the model provides mechanistic interpretability at multiple regulatory levels while delivering predictive performance close to that of a well-regularized linear baseline. This combination of accuracy and transparency addresses the key limitations of conventional deep learning models, which often lack interpretability and require large training cohorts that are rarely available in clinical settings [17].

A central strength of MiracleNet lies in its ability to identify robust and biologically meaningful biomarkers across miRNAs, genes, and pathways. By selecting biologically conserved miRNA–target pairs for training, the model leverages evolutionarily constrained interactions that link prioritized miRNAs to functionally relevant regulation [45]. The model revealed a substantial overlap in the top-ranked miRNAs between the cf and ev datasets, indicating shared relapse-associated regulatory programs detectable across liquid biopsy compartments. The concordance with classical differential survival analysis further supports the validity of these features, with some miRNAs independently prioritized by both approaches. At the gene level, MiracleNet consistently highlighted members of central oncogenic signaling pathways, including components of the RAS–MAPK and PI3K/AKT axes, as well as ubiquitin- and proteasome-related genes. These genes represent core nodes in pathways known to influence tumor proliferation, survival, and therapeutic resistance [34,35]. Pathway-level analysis revealed coherent enrichment of FGFR- and EGFR-related signaling modules, DNA replication and checkpoint regulation, and immune and inflammatory processes, all of which are well-established contributors to NSCLC progression and relapse [46,47]. Moreover, TGFβ–WNT pathways seem to be involved, as previously demonstrated by our study [14] and confirming the role of TGFβ expression and the risk of relapse in NSCLC patients [48].

A notable aspect of our findings is the overlap and complementarity between cf- and ev-derived miRNA signatures. Fourteen miRNAs were shared among the top-ranked features in both compartments, indicating a reproducible core regulatory program associated with relapse risk that is detectable across liquid biopsy modalities. These shared miRNA capture both mechanisms of tumor progression by miRNA dysregulation and are known to play a role in NSCLC, as discussed in Biological Relevance of Important Features. Beyond this shared signature, each compartment contributed distinct miRNAs, suggesting that cf- and ev-miRNAs capture partially distinct aspects of tumor and host biology: ev-miRNAs may reflect more tumor-specific vesicular communication, whereas cf-miRNAs may represent a broader mixture of tumor-derived and systemic physiological signals [49,50]. Together, the shared and compartment-specific miRNAs highlight the complementary nature of cf and ev measurements and show potential benefits of integrating both sources for comprehensive biomarker discovery.

The complementary analyses introduced in the “Nested evaluation of unimodal MiracleNet models” to “Multimodal cf + ev integration” sections support and extend our main findings. Under a stricter nested evaluation protocol, the pathway-only MiracleNet produces estimates consistent with the original protocol but with tighter standard deviations, indicating that the spread is not driven by leakage between hyperparameter selection and final scoring. Augmenting the network with clinically routine covariates (sex and clinical stage) raised the mean ev-miRNA test C-index to 0.600, approaching the penalized Cox baseline at 0.65 and confirming that integrating clinical information is constructive in the ev compartment. Multimodal cf + ev fusion produced a modest improvement over age-only unimodal models but did not exceed the ev + clinical configuration on the present data, suggesting that on this cohort the addition of clinical covariates contributes more than cross-compartment fusion. We interpret these analyses as evidence that MiracleNet’s value lies in its layered biological interpretability rather than in any specific advantage on raw predictive performance: a well-regularized linear baseline remains highly competitive, but only MiracleNet exposes importances at the miRNA, gene, and pathway levels jointly, and the consistency of those importances across cf- and ev-miRNAs (see Biological Relevance of Important Features) suggests that the layered representations are stable rather than artifacts of overfitting.

Our study also highlights the advantages of biologically informed architectures in the context of limited sample sizes. The constrained network structure reduces overfitting risk while preserving interpretability, allowing the model to uncover reproducible molecular patterns despite the modest cohort size. Nevertheless, certain limitations remain. Although our cohort is relatively large for miRNA-based NSCLC studies, further validation on independent datasets is necessary to confirm the generalizability of the identified biomarkers. Additionally, our model integrates age as the sole clinical variable; incorporating more extensive clinical data, radiomics, or additional omics layers could enhance predictive power. Extending the framework to multimodal or graph-based biologically informed neural networks represents a promising direction for future work. A formal leave-one-institution-out evaluation was not performed in this revision because per-site DFS event counts are small enough at the smallest contributing site to make site-held-out C-index estimates unstable and because a site-aware batch-effect assessment is a prerequisite that itself fell outside the revision window; we have addressed the underlying evaluation reliability concern through the nested cross-validation protocol introduced for the additional analyses. A formal learning-curve analysis is similarly left for follow-up work on expanded cohorts.

In summary, MiracleNet demonstrates that integrating biological prior knowledge into neural network architectures enables accurate relapse prediction while uncovering biologically coherent and clinically meaningful biomarker signatures in resected NSCLC. By linking miRNAs to their target genes and pathways, the model provides a mechanistically grounded view of relapse-associated processes and represents a promising step toward interpretable, clinically relevant artificial intelligence tools in oncology.

## Data Availability

The open-source code for reproducing the results of this study and for implementing the MiracleNet architecture is available at https://github.com/rashikajakhmola/Project-MIRACLE.
