# Supplementary material for: MiracleNet: A Biologically Interpretable Machine Learning Model for Resected Non-small-cell Lung Cancer
Source: Comput Struct Biotechnol J. 2026 Jul 14;35(1):0145. doi: 10.34133/csbj.0145 (PMC13365569; doi:10.34133/csbj.0145)
Supplement: Supplementary 1 — Appendix Tables S1 to S6 [file csbj.0145.f1.pdf]

A MiracleNet code implementation

Open-source code to reproduce the result of this study and to implement the MiracleNet architecture is available at https://github.com/rashikajakhmola/Project-MIRACLE.

To use the framework, data is added via PyTorch tensors and available hyperpameters of the `omics_net` class, which derives from the `torch.nn.Module` class, include the following architectural hyperparameters:

`in_nodes` Dimensionality of the observed features

`gene_nodes` Number of genes in the first hidden layer

`pathway_nodes` Number of pathways in the second hidden layer

`hidden_nodes` Tuple indicating the respective number of nodes in the third and fourth fully-connected hidden layers

The `forward` method accepts the following data inputs.

x_1 tensor of omics inputs

x_2 tensor of clinical data features

`gene_idx` predefined sparse masking matrix mapping input indices to genes

`pathway_idx` predefined sparse masking matrix mapping gene indices to pathways

`Drop_Rate` dropout rate

B Supplementary Tables

Table 4: Test-set performance on the cf-miRNA cohort across 10 random stratified 80/20 train–test splits. Values denote mean test C-index ± standard deviation and the best test C-index observed over the 10 runs.

**Model**	**Mean test C-index (10 runs)**	**Best test C-index**
MiracleNet (Biological Only)	0.49 ± 0.09	0.60
MiracleNet (Biological + Hidden)	0.47 ± 0.09	0.65
Sparse Neural Network	0.52 ± 0.09	0.68
Fully Connected Neural Network	0.45 ± 0.07	0.60
Penalized Cox Linear Baseline	0.56 ± 0.09	0.73

Table 5: Nested test-set performance of MiracleNet variants with additional clinical covariates on the cf-miRNA cohort. The age-only model uses the full cf cohort ($n = 169$, 43 events); the variants with sex and stage use the subset of patients with non-missing sex / stage annotations ($n = 155$, 41 events).

**Clinical input**	**Mean test C-index**	**95% CI**	**Best run**
Age only	0.485 ± 0.086	$[0.432, 0.539]$	0.647
Age + sex + clinical stage	0.459 ± 0.107	$[0.393, 0.525]$	0.600
Age + sex + pathological stage	0.553 ± 0.097	$[0.493, 0.613]$	0.710

Table 6: Top-25 miRNAs ranked by normalized importance from the MiracleNet model (Biological Only variant), for the ev-miRNA and cf-miRNA cohorts.

	EV		CF	
**Rank**	**Importance**	**miRNA**	**Importance**	**miRNA**
1	3.6247	miR-203a	3.4987	miR-9
2	3.4232	miR-195	3.3378	miR-203a
3	3.3681	miR-17	3.3284	miR-497
4	3.2820	miR-9	3.2515	miR-16
5	3.1086	miR-22	2.5227	miR-132
6	3.0670	miR-16	2.4731	miR-107
7	2.8871	miR-532	2.3963	miR-330
8	2.8203	miR-182	2.3959	miR-424
9	2.7695	miR-142	2.3345	miR-101
10	2.7559	miR-96	2.1187	miR-145
11	2.6796	miR-424	2.0504	miR-17
12	2.5866	miR-186	2.0359	miR-29a
13	2.4806	miR-107	2.0205	miR-142
14	2.4785	miR-93	2.0179	miR-128
15	2.4651	miR-140	1.9990	miR-140
16	2.4340	miR-183	1.9712	miR-195
17	2.3607	miR-15b	1.9425	miR-7
18	2.2376	miR-30e	1.9274	miR-15a
19	2.1985	miR-181a	1.8253	miR-30d
20	2.1740	miR-32	1.8034	miR-1271
21	2.1534	miR-340	1.7986	miR-143
22	2.1025	miR-15a	1.7984	miR-340
23	2.0606	miR-181d	1.7676	miR-30e
24	2.0501	miR-30a	1.7583	miR-29b
25	2.0199	miR-101	1.7399	miR-93

Table 7: Top-25 target genes ranked by normalized importance from the MiracleNet model (Biological Only variant), for the ev-miRNA and cf-miRNA cohorts.

	EV		CF	
**Rank**	**Importance**	**Gene Symbol**	**Importance**	**Gene Symbol**
1	6.8065	T	6.6080	T
2	3.8569	UBA52	4.1213	UBB
3	3.7714	AR	3.6515	UBC
4	3.7456	UBB	3.3366	UBA52
5	3.4166	UBC	3.2687	AR
6	3.2761	NRAS	2.6187	CS
7	3.0268	GRB2	2.3568	F3
8	2.1830	F3	2.1641	SOS1
9	2.0070	CALM1	2.0446	SP1
10	1.8903	C3	2.0248	GRB2
11	1.8827	PSMB5	1.9808	NRAS
12	1.8682	KRAS	1.9483	UBA5
13	1.8459	NF1	1.9479	KRAS
14	1.6273	FGF2	1.8793	PIK3CA
15	1.5992	PC	1.6519	PTPN1
16	1.5890	MAPK1	1.4805	ME1
17	1.5738	PSMD1	1.4799	IST1
18	1.5577	TPR	1.4739	PPP2CA
19	1.5263	PSME3	1.4600	AKT3
20	1.4361	SOS1	1.4334	PSMF1
21	1.3852	PPP2CA	1.3357	HIST1H2BD
22	1.3630	PIK3R1	1.3352	MAPK1
23	1.3489	MAPK3	1.2882	FGF2
24	1.3293	SKP1	1.2212	C7
25	1.3288	FRS2	1.1825	SMC3

Table 8: Top-25 pathways ranked by normalized importance from the MiracleNet model (Biological Only variant), for the ev-miRNA and cf-miRNA cohorts.

	**EV**		**CF**	
**Rank**	**Importance**	**Pathway**	**Importance**	**Pathway**
1	0.4675	REACTOME_DOWNSTREAM_SIGNALING_OF_ACTIVATED_FGFR4	0.5201	REACTOME_SIGNALING_BY_FGFR_IN_DISEASE
2	0.4261	REACTOME_ANTIVIRAL_MECHANISM_BY_IFN_STIMULATED_GENES	0.4539	REACTOME_SIGNALING_BY_LIGAND_RESPONSIVE_EGFR_VARIAN
3	0.4248	REACTOME_CA_DEPENDENT_EVENTS	0.4180	REACTOME_VEGFR2_MEDIATED_VASCULAR_PERMEABILITY
4	0.4072	REACTOME_DOWNSTREAM_SIGNALING_OF_ACTIVATED_FGFR2	0.4105	REACTOME_DOWNSTREAM_SIGNALING_OF_ACTIVATED_FGFR2
5	0.3972	REACTOME_SIGNALING_BY_FGFR	0.4086	REACTOME_CDK_MEDIATED_PHOSPHORYLATION_AND_REMOVA
6	0.3882	REACTOME_SUMOYLATION_OF_CHROMATIN_ORGANIZATION_PROTEINS	0.4069	REACTOME_DOWNSTREAM_SIGNALING_OF_ACTIVATED_FGFR1
7	0.3814	REACTOME_EXTRA_NUCLEAR_ESTROGEN_SIGNALING	0.4026	REACTOME_SIGNALING_BY_VEGF
8	0.3780	REACTOME_G_PROTEIN_MEDIATED_EVENTS	0.4004	REACTOME_DNA_REPLICATION
9	0.3727	REACTOME_DAG_AND_IP3_SIGNALING	0.3912	REACTOME_SIGNALING_BY_TYPE_1_INSULIN_LIKE_GROWTH_FAC
10	0.3579	REACTOME_NEGATIVE_REGULATION_OF_FGFR2_SIGNALING	0.3870	REACTOME_ACTIVATION_OF_NF_KAPPAB_IN_B_CELLS
11	0.3568	REACTOME_NUCLEAR_ENVELOPE_BREAKDOWN	0.3797	REACTOME_HEDGEHOG_OFF_STATE
12	0.3464	REACTOME_TOLL_LIKE_RECEPTOR_4_TLR4_CASCADE	0.3686	REACTOME_G2_M_DNA_DAMAGE_CHECKPOINT
13	0.3310	REACTOME_OPIOID_SIGNALLING	0.3668	REACTOME_G_PROTEIN_BETA:GAMMA_SIGNALLING
14	0.3244	REACTOME_TRANSPORT_OF_MATURE_MRNAS_DERIVED_FROM_INTRONLESS_TRANSCRIPTS	0.362[illegible]	REACTOME_FRS_MEDIATED_FGFR1_SIGNALING
15	0.3232	REACTOME_RORA_ACTIVATES_GENE_EXPRESSION	0.3587	REACTOME_SHC_MEDIATED_CASCADE:FGFR4
16	0.3189	REACTOME_ACTIVATION_OF_GENE_EXPRESSION_BY_SREBF_SREBP	0.3514	REACTOME_MITOTIC_METAPHASE_AND_ANAPHASE
17	0.3176	REACTOME_FCERI_MEDIATED_NF_KB_ACTIVATION	0.3431	REACTOME_SIGNALING_BY_EGFR_IN_CANCER
18	0.3171	REACTOME_SUMOYLATION_OF_TRANSCRIPTION_COFACTORS	0.3426	REACTOME_DNA_DAMAGE_BYPASS
19	0.3149	REACTOME_VIRAL_MESSENGER_RNA_SYNTHESIS	0.3381	REACTOME_SIGNALING_BY_NTRK3_TRKC
20	0.3136	REACTOME_SIGNALING_BY_NOTCH1_IN_CANCER	0.3345	REACTOME_DAP12_INTERACTIONS
21	0.3122	REACTOME_SIGNALING_BY_FGFR_IN_DISEASE	0.3316	REACTOME_GLYCOLYSIS
22	0.3117	REACTOME_MAPK6_MAPK4_SIGNALING	0.3306	REACTOME_INTERLEUKIN_2_FAMILY_SIGNALING
23	0.3104	REACTOME_PKA_MEDIATED_PHOSPHORYLATION_OF_CREB	0.3246	REACTOME_SIGNALING_BY_FGFR
24	0.3100	REACTOME_REGULATION_OF_PTEN_STABILITY_AND_ACTIVITY	0.3216	REACTOME_DOWNSTREAM_SIGNALING_OF_ACTIVATED_FGFR4
25	0.3073	REACTOME_SHC_MEDIATED_CASCADE:FGFR1	0.3097	REACTOME_SHC_MEDIATED_CASCADE:FGFR1

Table 9: Functional enrichment of top-25 miRNAs (cf and ev) using TAM 2.0 (miEAA). Only FDR-significant terms (adjusted $p < 0.05$) are shown in bold.

Group	Category	Subcategory	Enrichment	P-value	P-adjusted	Q-value	Exp.	Obs.	miRNAs / Precursors
**CF**	**Function (TAM 2.0)**	**Apoptosis**	**over-represented**	**0.0000473**	**0.0050172**	**0.0050172**	**6.67717**	**16**	**hsa-mir-101-1; hsa-mir-132; hsa-mir-143; hsa-mir-145; hsa-mir-15a; hsa-mir-16-1; hsa-mir-17; hsa-mir-195; hsa-mir-203a; hsa-mir-29a; hsa-mir-29b-1; hsa-mir-30e; hsa-mir-424; hsa-mir-497; hsa-mir-7-1; hsa-mir-9-1**
**CF**	**Function (TAM 2.0)**	**Inflammation**	**over-represented**	**0.0021647**	**0.0382434**	**0.0382434**	**7.05512**	**14**	**hsa-mir-101-1; hsa-mir-107; hsa-mir-132; hsa-mir-140; hsa-mir-142; hsa-mir-143; hsa-mir-145; hsa-mir-17; hsa-mir-203a; hsa-mir-29a; hsa-mir-424; hsa-mir-7-1; hsa-mir-9-1; hsa-mir-93**
**CF**	**Function (TAM 2.0)**	**Immune Response**	**over-represented**	**0.0010229**	**0.0293951**	**0.0293951**	**5.79528**	**13**	**hsa-mir-101-1; hsa-mir-107; hsa-mir-132; hsa-mir-140; hsa-mir-143; hsa-mir-15a; hsa-mir-16-1; hsa-mir-17; hsa-mir-203a; hsa-mir-29a; hsa-mir-424; hsa-mir-9-1; hsa-mir-93**
**CF**	**Function (TAM 2.0)**	**Cell Cycle**	**over-represented**	**0.0015062**	**0.0319321**	**0.0319321**	**5.22835**	**12**	**hsa-mir-107; hsa-mir-140; hsa-mir-143; hsa-mir-145; hsa-mir-15a; hsa-mir-16-1; hsa-mir-17; hsa-mir-195; hsa-mir-29b-1; hsa-mir-424; hsa-mir-497; hsa-mir-9-1**
**CF**	**Function (TAM 2.0)**	**Cell Death**	**over-represented**	**0.0034021**	**0.0499777**	**0.0499777**	**4.91339**	**11**	**hsa-mir-128-1; hsa-mir-143; hsa-mir-145; hsa-mir-16-1; hsa-mir-17; hsa-mir-203a; hsa-mir-29a; hsa-mir-29b-1; hsa-mir-30d; hsa-mir-497; hsa-mir-7-1**
**CF**	**Function (TAM 2.0)**	**Cell Proliferation**	**over-represented**	**0.0042434**	**0.0499777**	**0.0499777**	**5.03937**	**11**	**hsa-mir-140; hsa-mir-143; hsa-mir-145; hsa-mir-15a; hsa-mir-16-1; hsa-mir-17; hsa-mir-203a; hsa-mir-29a; hsa-mir-29b-1; hsa-mir-9-1; hsa-mir-93**
**CF**	**Function (TAM 2.0)**	**Hormone-mediated Signaling Pathway**	**over-represented**	**0.0011092**	**0.0293951**	**0.0293951**	**3.65354**	**10**	**hsa-mir-101-1; hsa-mir-128-1; hsa-mir-132; hsa-mir-15a; hsa-mir-16-1; hsa-mir-17; hsa-mir-29a; hsa-mir-30d; hsa-mir-7-1; hsa-mir-9-1**
**CF**	**Function (TAM 2.0)**	**Chemosensitivity Of Tumor Cells**	**over-represented**	**0.000724**	**0.0293951**	**0.0293951**	**0.503937**	**4**	**hsa-mir-107; hsa-mir-140; hsa-mir-145; hsa-mir-16-1**
**CF**	**Function (TAM 2.0)**	**Smooth Muscle Cell Senescence**	**over-represented**	**0.0038127**	**0.0499777**	**0.0499777**	**0.125984**	**2**	**hsa-mir-143; hsa-mir-145**

Continued on next page

Group	Category	Subcategory	Enrichment	P-value	P-adjusted	Q-value	Exp.	Obs.	miRNAs / Precursors
**EV**	**Function (TAM 2.0)**	**Immune Response**	**over-represented**	**0.00000585**	**0.000532**	**0.000532**	**5.79528**	**16**	**hsa-mir-101-1; hsa-mir-107; hsa-mir-140; hsa-mir-15a; hsa-mir-16-1; hsa-mir-17; hsa-mir-181a-1; hsa-mir-181d; hsa-mir-186; hsa-mir-203a; hsa-mir-22; hsa-mir-30a; hsa-mir-424; hsa-mir-532; hsa-mir-9-1; hsa-mir-93**
**EV**	**Function (TAM 2.0)**	**Circadian Rhythm**	**over-represented**	**0.000146**	**0.0066295**	**0.0066295**	**1.38583**	**7**	**hsa-mir-107; hsa-mir-16-1; hsa-mir-181a-1; hsa-mir-181d; hsa-mir-182; hsa-mir-183; hsa-mir-96**
**EV**	**Function (TAM 2.0)**	**Apoptosis**	**over-represented**	**0.0011634**	**0.0352889**	**0.0352889**	**6.67717**	**14**	**hsa-mir-101-1; hsa-mir-15a; hsa-mir-15b; hsa-mir-16-1; hsa-mir-17; hsa-mir-181a-1; hsa-mir-182; hsa-mir-195; hsa-mir-203a; hsa-mir-30a; hsa-mir-30e; hsa-mir-424; hsa-mir-9-1; hsa-mir-96**
